# Rationale and design of a navigator‐driven remote optimization of guideline‐directed medical therapy in patients with heart failure with reduced ejection fraction

**DOI:** 10.1002/clc.23291

**Published:** 2019-11-14

**Authors:** Alexander J. Blood, Christina M. Fischer, Liliana E. Fera, Taylor E. MacLean, Katelyn V. Smith, Jacqueline R. Dunning, Joshua W. Bosque‐Hamilton, Samuel J. Aronson, Thomas A. Gaziano, Calum A. MacRae, Lina S. Matta, Ana A. Mercurio‐Pinto, Shawn N. Murphy, Benjamin M. Scirica, Kavishwar Wagholikar, Akshay S. Desai

**Affiliations:** ^1^ Cardiovascular Innovation Program Brigham and Women's Hospital Boston Massachusetts; ^2^ Cardiovascular Division Brigham and Women's Hospital Boston Massachusetts; ^3^ Massachusetts General Hospital Boston Massachusetts; ^4^ Research Information Science and Computing, Partners Healthcare Somerville Massachusetts

**Keywords:** clinical, clinical trials, coronary revascularization, computers in cardiovascular medicine, heart failure, pharmacology

## Abstract

Although optimal pharmacological therapy for heart failure with reduced ejection fraction (HFrEF) is carefully scripted by treatment guidelines, many eligible patients are not treated with guideline‐directed medical therapy (GDMT) in clinical practice. We designed a strategy for remote optimization of GDMT on a population scale in patients with HFrEF leveraging nonphysician providers. An electronic health record‐based algorithm was used to identify a cohort of patients with a diagnosis of heart failure (HF) and ejection fraction (EF) ≤ 40% receiving longitudinal follow‐up at our center. Those with end‐stage HF requiring inotropic support, mechanical circulatory support, or transplantation and those enrolled in hospice or palliative care were excluded. Treating providers were approached for consent to adjust medical therapy according to a sequential, stepped titration algorithm modeled on the current American College of Cardiology (ACC)/American Heart Association (AHA) HF Guidelines within a collaborative care agreement. The program was approved by the institutional review board at Brigham and Women's Hospital with a waiver of written informed consent. All patients provided verbal consent to participate. A navigator then facilitated medication adjustments by telephone and conducted longitudinal surveillance of laboratories, blood pressure, and symptoms. Each titration step was reviewed by a pharmacist with supervision as needed from a nurse practitioner and HF cardiologist. Patients were discharged from the program to their primary cardiologist after achievement of an optimal or maximally tolerated regimen. A navigator‐led remote management strategy for optimization of GDMT may represent a scalable population‐level strategy for closing the gap between guidelines and clinical practice in patients with HFrEF.

## 1 INTRODUCTION

Although optimal pharmacological therapy for heart failure with reduced ejection fraction (HFrEF) is carefully scripted by treatment guidelines, many eligible patients are not treated with guideline‐directed medical therapy (GDMT) in clinical practice.^1^ In data recently published from the CHAMP‐HF (Change the Management of Patients with Heart Failure) registry of ambulatory heart failure patients in the United States with HF and reduced EF, roughly one‐third of eligible patients were not receiving beta‐blockers (β‐blockers), one‐fourth were not receiving angiotensin‐converting enzyme inhibitors (ACEI), angiotensin receptor blockers (ARB) or angiotensin receptor‐neprilysin inhibitors (ARNI), and two‐thirds were not prescribed mineralocorticoid receptor antagonists (MRA). Amongst those receiving these therapies, the vast majority are dosed below guideline‐recommended targets, with only 1% of patients eligible for all classes of medication receiving target doses of all three medication classes. Since appropriate application of GDMT is associated with considerable reductions in heart failure‐associated morbidity and mortality, these data suggest a considerable opportunity for quality improvement.^2^

Although prescription or dose titration of GDMT may in some cases be limited by blood pressure, heart rate, renal function, or serum potassium, medical contraindications are not always apparent, suggesting that other factors may be responsible for the implementation gap. Possible alternative explanations include lack of familiarity with guideline recommendations, infrequent clinic‐based follow‐up, uncertainty regarding the value of dose titration, limited opportunity to make dose adjustments in the clinic setting, concerns about tolerability, a focus on arbitrary numerical values for discrete endpoints, opportunity costs to patients and physicians, and difficulty in implementing adequate laboratory surveillance.^3^ To overcome some of these barriers, we designed a strategy for remote optimization of GDMT on a population scale in patients with HFrEF leveraging nonphysician providers in a collaborative practice model. In this manuscript, we summarize the details of the design and implementation of this program, as well as preliminary enrollment data supporting the feasibility of this approach.

## 2 PROGRAM DESIGN

As part of a broader effort at quality improvement in population health, we launched the Virtual Heart Failure Clinic (VHFC) at Brigham and Women's Hospital in 2017. The overarching goal of the program is to systematically identify patients with heart failure and reduced ejection fraction who are longitudinally managed by Brigham and Women's Hospital providers and facilitate remote optimization of GDMT through a telephone‐based, navigator‐led approach. Eligible patients were identified through a search of electronic health records (EHRs), and included women and men ≥18 years of age with a diagnosis of chronic heart failure and left ventricular ejection fraction ≤40%. All patients had to have an established relationship with a cardiology provider at our center, defined by at least two previous visits including one within the 18 months prior to enrollment. Patients with end‐stage HF requiring inotropic support, mechanical circulatory support, transplantation, and those enrolled in hospice or palliative care were excluded. Detailed inclusion and exclusion criteria are summarized in Table 1.

**Table 1:** Key eligibility criteria

Inclusion criteria	Exclusion criteria
Age ≥ 18 year Documented heart failure Most recent echo documents EF ≤40 Seen twice by a BWH cardiologist with at least one visit in the last 18 months Reliable telephone access English speaking	End stage renal disease Active chemotherapy Receiving end of life care or life expectancy ≤1 year Any transplant (heart, kidney etc.) Currently listed or being evaluated for transplant IV inotrope use Use of a ventricular assist device (VAD) or CardioMEMs device Frailty/fall risk Acute decompensated heart failure Evidence or history of medication nonadherence

We developed a search strategy to identify suitable patients with heart failure from the EHR. The initial approach used billing codes to derive a set of coded inclusion and exclusion criteria to identify patients with likely heart failure and creation of a data mart of all patients who met these criteria since 1990. A clinical subject matter expert then reviewed the medical charts for 250 patients randomly selected from the data mart. This review created a gold standard which was used to train a statistical model to predict the presence or absence of HF at a positive predictive value threshold of 90%. We further refined this data‐mart using natural language processing to identify patients who were most likely to meet the eligibility criteria.^4^ Baseline characteristics of the patients recruited into the study are included in Table 2.

**Table 2:** Pre‐intervention baseline characteristics

	Mean or no. (SD or %)
Age, year	64.99 (12.28)
Female sex	47 (29.56%)
Race	
African American	26 (16.35%)
NYHA class functional class	
I	51 (32.08%)
II	90 (56.60%)
III	18 (11.32%)
IV	0 (0.00%)
Clinical characteristics	
Systolic blood pressure, mm Hg	129.94 (15.35)
Diastolic blood pressure, mm Hg	71.63 (10.19)
Heart rate, bpm	72.52 (12.68)
LVEF	32.30 (6.85)
Weight, lbs.	197.99 (43.63)
Serum creatinine, mg/dL	1.13 (0.50)
eGFR, mL/min/1.73 m^2^	57.00 (8.41)
Medical history	
Atrial fibrillation	52 (32.70%)
Diabetes	43 (27.04%)

**Figure 1:**
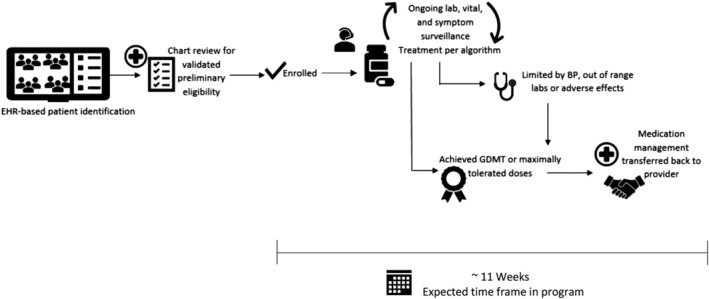
Study design and workflow. BP, blood pressure; EHR, electronic health record; GDMT, guideline‐directed medical therapy

Patients identified through the EHR‐based search were contacted via phone by a navigator who completed a medication reconciliation and verification of eligibility for participation in the remote optimization program. Treating providers were then approached for consent to adjust medical therapy according to a sequential, stepped titration algorithm modeled on the current ACC/AHA HF Guidelines. The program was approved by the institutional review board at Brigham and Women's Hospital with a waiver of written informed consent. All patients provided verbal consent to participate. Patients and providers who declined to participate in the remote optimization program served as a reference group. This workflow is detailed in Figure 1.

### 2.1 Drug titration

**Figure 2:**
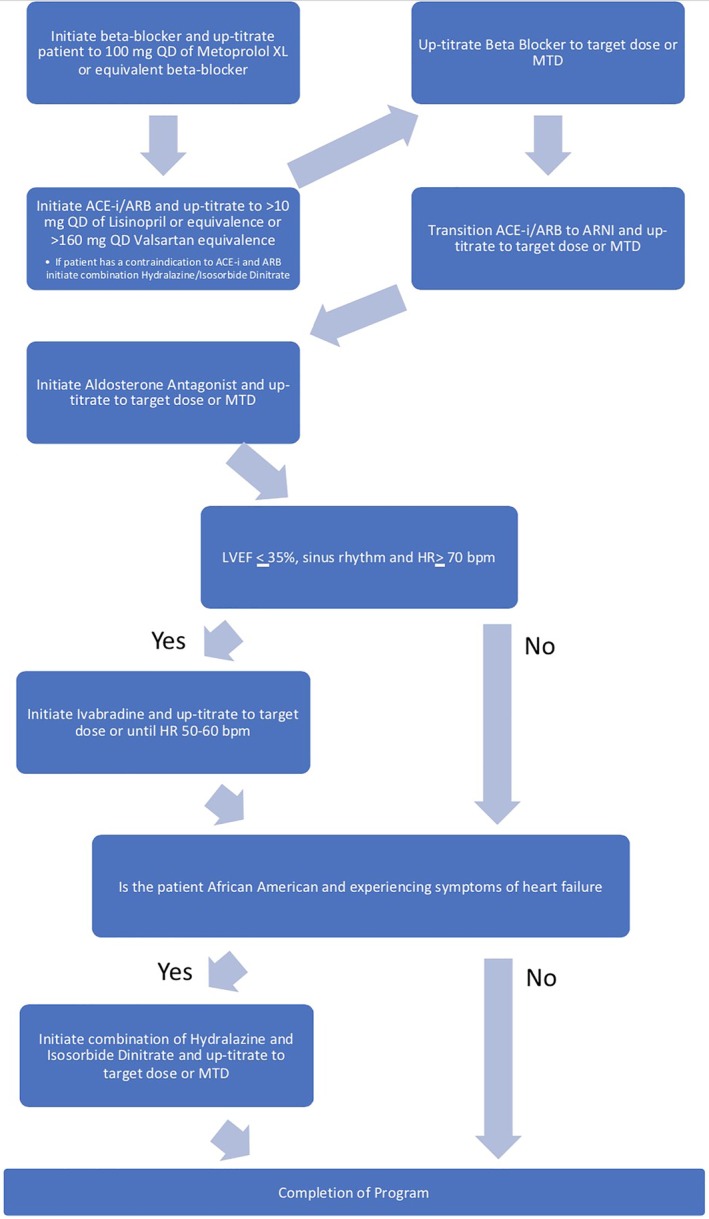
Medication titration algorithm. ACEI, angiotension converting enzyme inhibitor; ARB, angiotensin receptor blocker; ARNI, angiotensin receptor‐neprilysin inhibitors; bpm, beats per minute; HR, heart rate; LVEF, left ventricular ejection fraction; MTD, maximum tolerated dose; QD, daily

For patients enrolled in the remote optimization program, medication titration was overseen by pharmacists practicing under a Collaborative Drug Therapy Management (CDTM) agreement. Protocols for the initiation, discontinuation, and titration of β‐blockers, ACEI, *angiotensin II receptor blockers*, ARNI, aldosterone antagonists, sinus node inhibitors, hydralazine, and isosorbide dinitrate were developed by a team of pharmacists, nurses, general cardiologists, and cardiology heart failure specialists to approval through multidisciplinary review at the BWH Pharmacy and Therapeutics Committee. These protocols were heavily based on published guidelines and formed the basis of the CDTM agreement. When the sequence of introduction of therapy was not explicitly defined in guidelines, our team made these decisions based on the ACC expert consensus statement and clinical practice.^5^, ^6^ The CDTM agreement allowed pharmacists to initiate, discontinue, and titrate all medication classes outlined in Figure 2. We developed a software application to generate a HF medication change based on patient‐specific information and to longitudinally monitor each participant's progress through the algorithm and document clinical, laboratory, and vital sign information. Basic patient demographic, laboratory, medication, and medical history data were housed in a Microsoft SQL Server 2017 database. These data interacted through an application programming interface (API) server build using Java (v1.8), Spring Boot (v1.5.14), and Hibernate (v5.2.9). This API was used to persist patient information and populate a treatment recommendation algorithm that was implemented using JavaScript (es2015). JavaScript was selected given its flexibility in allowing for iterative algorithm modifications. To maintain comprehensive audit logs, the API server used Hibernate Envers (v5.2.9) to manage all database interactions. The user interface was packaged as Windows and Mac desktop clients using ReacjJS (v15.6.2) and Electron (v1.4.13), which allowed for use from team member workstations. In addition to treatment decision making, the application also provided a scheduling tool for team members to coordinate patient follow‐up telephone calls and laboratory testing, a messaging tool that allowed multidisciplinary team members to coordinate individual patient care tasks and other patient management capabilities. All aspects of the application complied with the Health Insurance Portability and Accountability Act of 1996 and institutional requirements.

A navigator was assigned to act as the primary interface with the patient. The navigator was typically a bachelor's level or master's level trained individual who did not have formal clinical training but was qualified to approach patients and solicit basic information about demographics and fundamental clinical data. Titration towards GDMT was conducted in a stepwise manner (Figure 2) by a pharmacist/navigator team, under the supervision of a nurse practitioner and HF cardiologist. Each algorithm‐derived titration step was passed from the pharmacist to the navigator. The navigator then facilitated medication adjustments by telephone and conducted longitudinal surveillance of laboratory values, blood pressure, and symptoms in accordance with approved protocols. This information was relayed back to the pharmacist through the EHR where the pharmacist then signed the prescription for the new medication under the CDTM agreement.

Medication initiation and titration orders were dictated by the algorithm (Figure 2). Titrations proceeded until patients reached the guideline‐directed target doses, reported intolerable symptoms, or met criteria for no further adjustment, which was generally dependent on blood pressure, serum potassium levels, and renal function (Table S1). Specific rules governing sequencing and titration of each drug class are provided in Appendix 1.

### 2.2 Follow‐up

Patients were considered to have graduated from the VHFC once they achieved the guideline directed or maximally tolerated dose of all guideline‐based medications for which there was an indication. At graduation, the management of the patient's heart failure medications was passed back to the patient's primary cardiologist. Patients were contacted again by phone 3 months after graduation from the program to complete a medication reconciliation and ensure there were no new side effects. Six months following graduation was the final follow‐up consisting of a medication reconciliation, laboratory surveillance, and assessment of NYHA class. The final follow‐up was conducted either by phone or by chart abstraction if the patient had a cardiology visit within 1 month of the scheduled final study follow‐up.

## 3 OUTCOMES

The primary goal of the intervention was to enhance the proportion of patients receiving >50% of guideline directed doses of GDMT at 3 months following initial contact in the remote medication optimization group compared with the reference group of patients who declined to participate in the medication titration intervention. Key safety outcomes of interest included the proportion of emergency department visits, hospitalizations, and deaths during study follow‐up in both groups.

## 4 STATISTICAL CONSIDERATIONS

As this study was organized as a quality improvement intervention rather than a clinical trial, no formal power calculation was performed. Based on anticipated recruitment, our sample targeted 1000 patients, baseline utilization of GDMT at >50% of target doses in 20% of patients, and projected enrollment of 25% of subjects in the remote medication optimization arm, we anticipate the study will provide >80% power to detect an absolute improvement of 10% in utilization of GDMT using this approach.

## 5 DISCUSSION

Optimization of GDMT has been associated with reductions in cardiovascular and heart failure morbidity and mortality in numerous clinical trials, registries, and meta‐analyses.^7^, ^8^, ^9^, ^10^, ^11^, ^12^, ^13^, ^14^, ^15^, ^16^, ^17^, ^18^, ^19^, ^20^, ^21^ However, clinicians frequently fail to implement guideline directives in practice.^22^ These gaps in care have been attributed to numerable factors, such as inertia, reluctance to increase medication burden, cost, lab monitoring, requirements for insurance pre‐authorization, and lack of knowledge about rapidly evolving evidence.^23^, ^24^ There is a substantial opportunity for meaningful improvement in clinical outcomes amongst HF patients, however, the 2013 AHA/ACC guidelines for the management of heart failure encourage strategies to close the gap between current practice and guideline recommendations.^1^

Even when clinicians apply HF medications as directed by guidelines, medications are frequently not dosed to guideline‐recommended targets, and infrequent clinic‐based contact means that the medical regimen evolves over a protracted time interval, with many months lapsing between medication titration. Given that the benefits associated with deployment of GDMT are often seen early, this may reflect a missed opportunity to improve patient outcomes.^25^, ^26^, ^27^ Moreover, deployment of invasive strategies for HF including ICD and/or CRT is intended to follow on medical optimization, since this therapy may in many cases result in reverse remodeling that can lead to improvements in EF over time and obviate the need for device therapy.^28^, ^29^ Unfortunately, data suggests that most patients who receive ICD or CRT do not optimize GDMT prior to device implantation, reflecting another missed opportunity for these patients.^30^ These gaps in care are associated with significant mortality for patients with HFrEF.^31^

A number of approaches to enhance GDMT utilization and address gaps in implementation have been explored. Research initiatives aimed at understanding and addressing gaps in care (summarized in Table 3) have failed to consistently and reproducibly change behavior and impact outcomes. Educational strategies focused on patients and providers to emphasize the value of guideline‐driven care are clearly important, but the ability of these initiatives to rapidly drive changes in clinical practice is unclear.^31^ Although traditional multidisciplinary HF disease management programs do achieve higher utilization and less discontinuation of GDMT, such programs are not accessible to the vast majority of HF patients, and rates of optimal GDMT utilization in these clinics still falls well below guideline‐recommended targets.^6^, ^35^, ^45^, ^46^ However, research suggests that improving upon current rates of GDMT is possible and innovative approaches to improving optimal rates of adoption and goal dosing have shown promise.^36^, ^47^, ^48^ Early experience suggests that integration of pharmacists in collaborative practice agreements may facilitate optimizing medical therapy in HFrEF patients, but systematic exploration of these efforts at scale has not been completed yet and have not incorporated the use of nonclinician navigators nor expanded to include full complement of GDMT for HFrEF.^33^, ^49^, ^50^

**Table 3:** Comparison of clinical trials implementing strategies to improve GDMT utilization in congestive heart failure

Study	Study size	Study population (country)	Summary of intervention	Duration (frequency of intervention)	Summary outcomes
Retrospective cohort					
Jain et al^32^	265	Outpatient Cardiology (UK)	Pharmacist and RN‐led CHF Education and Medication Titration	30 months (variable)	Improved rates of GDMT (57%‐11%, *P* < .001) Improved rates of target‐dose therapy for GDMT (18%‐57%, *P* < .001)
Bhat et al^33^	148	Outpatient (United States)	Pharmacist‐managed Medication Titration Assistance Clinic	12 months (variable)	Increased rates of target or maximum‐tolerated ACEI/ARB and β‐blocker in those not initially at optimal dosing in pharmacist‐directed vs general cardiology clinics (64% vs 40%, data not provided)
Balakumaran et al^34^	61	Outpatient (United States)	Nurse‐led Clinic focused on implementing GDMT	24 months (every 2 weeks)	Increased number of GDMT therapies (2.31 ± 0.76‐2.74 ± 0.66, *P* < .001) and target doses (0.54 ± 0.79‐1.52 ± 1.1, *P* < .001) with an improvement in LVEF (21.8 ± 7.8‐36.2 ± 14.3, *P* < .001) and a reduction in heart failure hospitalizations 26‐8, *P* < .001
Prospective cohort					
Hickey et al^35^	280	CHF Hospitalization (Australia)	A structured medication titration plan at the time of hospital discharge	6 months (variable)	Improvements in achieving target doses of β‐blockers (38%‐54%, *P* = .013) and ACEI/ARB (34%‐54% *P* = .001)
Fonarow et al^6^	34, 810	Outpatient Cardiology Practices (United States)	Clinical decision support tools; Structured improvement strategies; Chart audits with feedback	24 months (baseline, 6, 12, 18, and 24 months)	Increases in β‐blocker (7.4%, 6.6‐8.2,) aldosterone antagonist (27.4%, 24.3‐30.6), CRT‐P/CRT‐D (30.9%, 27.2‐34.5), ICD/CRT‐D (30.3%, 28.8‐31.8), and CHF education (9.1%, 7.8‐10.4) all *P* < .001
Braun et al^36^	208	Outpatient Family Physicians (Germany)	Computer‐based reminder system; Provider Education	20 months (8 months pre‐ and 12 months post‐ intervention)	No significant difference in GDMT prescription rates (*P* values ranged from 0.09 to 0.98) with an increase in the rate of evidence‐based β‐blocker prescription (12.3% ‐ > 58.6%, *P* = .03)
Murphy et al^49^	100	CHF Hospitalization (United States)	Patient education; Outpatient Pharmacist Appointment	1 month (variable)	No significant difference in 30‐day readmission rates (ARR 24% ‐ > 18%, *P* = .238)
Randomized controlled trial					
Gattis et al^51^	181	Outpatient Clinics (United States)	Medication recommendations; CHF Medication Education	6 months (2, 12, and 24 weeks)	Reduction in mortality and nonfatal CHF hospitalization (OR 0.22, 0.07‐0.65, *P* = .005) Closer to target‐dose for ACE‐I therapy in intervention Fraction, 25th, 75th percentile (1, 0.5, 1) vs control (0.5, 0.188, 1) *P* < .001
Bouvy et al^37^	152	CHF Hospitalization (The Netherlands)	Medication History; CHF Medication Education; Medication Compliance; Liaison with GP	6 months (monthly)	No difference in death or hospitalizations 1.1 (0.5‐2.2) Decrease in days without dosing 0.3 (0.2‐0.6)
Tsuyuki et al^54^	276	CHF Hospitalization (Canada)	Pharmacist or nurse provided CHF Medication Education; Monthly follow‐up; Adherence aids	6 months (at 2 weeks and monthly)	No difference in medication adherence Reduction in CV emergency department visits (*P* = .30) and hospitalization days (*P* = .003)
Gwadry‐Sridhar et al^38^	134	CHF Hospitalization (Canada)	Inpatient CHF Medication and lifestyle Education	12 months (single episode)	No difference in medication compliance rates (RR 0.78, 0.33‐1.89 for ACE‐I/ARB) or death, ED visit, or re‐hospitalization (HR 0.85, 0.55‐1.30)
Murray et al^39^	314	Outpatient General Medicine and Cardiology (United States)	Medication History; CHF Medication Education; Medication Compliance	12 months (variable)	Reduction in hospitalization and ED visits (HR 0.82, 0.73‐0.93) No sustained difference in medication adherence (3.9% ARR, −5.9 to +6.5%)
Holland et al^40^	291	CHF Hospitalization (UK)	Home visits by pharmacist with Medication review; CHF Medication and Lifestyle Education	6 months (2 home visits within 2–8 weeks of discharge)	No difference in hospital admissions (rate ratio 1.15, 0.89‐1.48) or death (Log rank *P* = .51)
Eggink et al^41^	85	CHF Hospitalization (The Netherlands)	Medication reconciliation by a pharmacist prior to discharge	1 month (single episode)	Decrease in discrepancies and prescription errors (RR 0.42, 0.27‐0.66)
Korajkic et al^42^	70	Outpatient Clinics (Australia)	Pharmacist led CHF Medication and Lifestyle education with diuretic dosing	3 months (single episode)	Increased diuretic adjustment (0.9 ± 0.1 vs 0.3 ± 0.08, *P* = .006) with a reduction in hospital readmissions for volume overload in the intervention group (14% vs 31%, *P* = .04)
Lowrie et al^43^	2169	Outpatient Clinics (UK)	30‐minute pharmacist appointment for CHF Medication Education and optimization	24 months (baseline +3‐4 weekly consultations)	No difference in death, CV or all‐cause hospitalizations (HR 0.97, 0.83‐1.14, *P* = .72) Improvements in optimal doses of ACEI and β‐blocker therapy (OR 2.26, 1.64‐3.10, *P* < .001)
Meta‐analysis					
Driscoll et al^44^	1684	Outpatient (Multinational)	Nurse‐led titration of GDMT medications	N/A	Lower all‐cause (RR 0.8, 0.72‐0.88) and CHF (0.51, 0.36‐0.72) hospitalization rates, all‐cause mortality (RR 0.66, 0.48‐0.92), and improved rates of optimal doses of GDMT (RR 1.99, 1.61‐2.67)

Since algorithms for initiation, titration, and even discontinuation of medical therapy for HF are detailed in major society guidelines, there may be an opportunity to improve appropriate application of GDMT on a population scale by leveraging nonphysician providers to supplement the work of dedicated HF clinicians. Such collaborative practice models may enable more rapid evolution of the medical regimen outside the clinic setting, while muting practice variation with regard to drug titration and laboratory surveillance. As well, they may introduce economies of scale with regard to insurance authorization for costly medications and facilitate more rapid translation of new guideline‐directives to clinical practice. Utilizing navigators as well as pharmacists in the context of collective practice agreements may help clinicians to care for larger numbers of patients in a standardized and cost‐effective manner.^44^

In summary, we propose to test the efficacy and safety of a collaborative, remote management strategy for medication optimization as a means of closing the implementation gap between guidelines and clinical practice. We anticipate that demonstration of the preliminary effectiveness of this approach for enhancing utilization of GDMT amongst patients with HF and reduced EF in clinical practice may help provide support for future prospective, randomized investigations of this approach in clinical practice.

## Supporting information


**Table S1**

## Appendix 1 A.

*β‐Blocker titration*: Patients who were naïve to β‐Blocker therapy were initiated on metoprolol succinate at a starting dose of 12.5 mg daily. Patients who were on an evidence based β‐blocker began titrations from their current dose, unless there was evidence or concern that the patient would not tolerate further titration. All nonevidence based β‐blocker doses were converted to equivalent doses of metoprolol succinate, in the absence of contraindications. Patients who were currently taking sotalol at any dose level were considered to have met their maximally tolerated dose.

*ACEI and ARB titration*: Patients who were naïve to ACEI or ARB therapy were initiated on lisinopril at a starting dose of 2.5 mg QD, all other patients began titrations from their current dose of their existing ACEI or ARB, unless there is evidence or concern that the patient would not tolerate further titration. Patients who experienced an intolerable cough on an ACEI were converted to an equivalent dose of losartan. Lab surveillance was conducted 10 ± 3 days after each medication titration. An increase in serum creatinine greater than 30% from baseline or an increase in potassium greater than 15% from baseline prompted repeat lab surveillance in 7‐10 days. In patients who could not tolerate the lowest dose of an ACEI or a dose reduction was indicated due to systolic blood pressures below 90 mmHg or symptomatic hypotension, the dose of β‐blocker was preferentially reduced and the patient was re‐evaluated in 1 week. If the patient was still unable to tolerate these doses, the ACEI or ARB was removed and the patient was re‐evaluated in 1 week. If symptoms resolved and/or systolic blood pressure returned to ≥95 mmHg the β‐blocker was resumed at the previously tolerated dose. If symptoms did not resolve or systolic blood pressure did not return to ≥95 mmHg adjustment of the β‐blocker continued until no further adjustment was indicated per Table S1.

*ARNI titration*: Patients who were eligible for ARNI therapy per the current ACC/AHA HF Guidelines and who proved tolerability to an ACEI or ARB at daily doses of >10 mg or 160 mg, respectively, were transitioned to a middose (49/51 mg) ARNI. Patients who met the guideline criteria for consideration of ARNI therapy but who could not tolerate an ACEI or ARB at daily doses of >10 mg or 160 mg, respectively, or who had an eGFR of <30 were initiated on low dose (24/26 mg) ARNI therapy. Lab surveillance was conducted 10 ± 3 days after each medication titration. An increase in serum creatinine greater than 30% from baseline or an increase in potassium greater than 15% from baseline prompted repeat lab surveillance in 7‐10 days.

*MRA titration*: Patients who were naïve to MRA therapy were initiated on spironolactone at a starting dose of 25 mg QOD. All other patients began titrations from their current dose of either spironolactone or eplerenone. Patients who experienced sexual side effects at any dose of spironolactone were converted to the equivalent dose of eplerenone and re‐evaluated. Lab surveillance was conducted 7‐10 days after each dose adjustment.

*Ivabradine titration*: Ivabradine titrations began at 2.5 mg QD and occurred every 4 weeks for patients whose heart rate remained elevated despite receiving all other guideline indicated therapy.

*Hydralazine and isosorbide dinitrate titration*: Titration of hydralazine and isosorbide dinitrate was done in tandem from a starting dose of 25/20 mg. Titrations proceeded in 25 mg and 10 mg increments, respectively, one additional titration of hydralazine was conducted to attain the guideline directed dosing. For patients who were already receiving monotherapy, titrations began with the initiation and titration of the absent medication class until the patient achieved a dose level from which tandem titrations can proceed.

RR, relative risk (95% confidence interval unless otherwise specified); ARR, absolute risk reduction; HR, hazard ratio. References 6, 32, 34, 36, 37, 38, 39, 40, 41, 42, 43, 44, 45, 47, 49, 51, 52, 53, 54, 55
