# Supplementary material for: Rationale and design of a navigator‐driven remote optimization of guideline‐directed medical therapy in patients with heart failure with reduced ejection fraction
Source: Clin Cardiol. 2019 Nov 14;43(1):4–13. doi: 10.1002/clc.23291 (PMC6954374; doi:10.1002/clc.23291)
Supplement: Supplementary file 1 — Table S1 [file CLC-43-4-s001.docx]

| **Supplementary Table 1** | ACEi/ARB | β-Blocker | ARNI | ARNI* | MRA | Ivabradine | Hydralazine/Nitrates |
| --- | --- | --- | --- | --- | --- | --- | --- |
| Initiation | K: < 5.0  SCr: < 3.0  SBP: ≥ 95 | SBP: ≥ 95  HR: ≥ 50 | K: ≤ 5.4  SCr: < 3.0  SBP: ≥ 95 | K: ≤ 5.4  SCr: < 3.0  SBP: ≥ 105 | K: < 5.0  SCr: ≤2.5 in men or ≤ 2 in women  eGFR:> 30  SBP: ≥ 95 | SBP: ≥ 95  HR: ≥ 70 | SBP: ≥ 95 |
| Up Titration | K: < 5.5  SCr: < 3.0 and < 50% increase from baseline  SBP: ≥ 95 | SBP: ≥ 95  HR: ≥ 50 | K: < 5.4  SCr: < 3.0 and < 50% increase from baseline  SBP: ≥ 95 | K: < 5.4  SCr: < 3.0 and < 50% increase from baseline  SBP: ≥ 105 | K: < 5.5  SCr: ≤2.5 in men or ≤ 2 in women and < 50% increase from baseline  SBP: ≥ 95 | SBP: ≥ 95  HR: >60 | SBP: ≥ 95 |
| Down Titration | K: ≥ 5.6  SCr: ≥ 3 or ≥ 50% increase from baseline  SBP: <90 | SBP: <90  HR < 50 | K: ≥ 5.6  SCr: ≥ 3 or ≥ 50% increase from baseline  SBP: <90 | K: ≥ 5.6  SCr: ≥ 3 or ≥ 50% increase from baseline  SBP: <90 | K: ≥ 5.6  SCr: >2.5 in men or >2 in women or ≥ 50% increase from baseline  eGFR:≤ 30  SBP: ≥ 90 | SBP: < 90  HR:< 50 | SBP: < 90 |
| No Further Adjustment | K: 5.5  SBP: ≥ 90 <95 | SBP: ≥ 90 <95 | K: 5.5  SBP: ≥ 90 <95 | K: 5.5  SBP: ≥ 90 <105 | K: 5.5  SBP: ≥ 90 <95 | SBP: ≥ 90 <95  HR ≤60 ≥50 | SBP: ≥ 90 <95 |
